# Effect of a health education program on teachers’ knowledge and attitude regarding bacterial antibiotic resistance among high school teachers in Koya District

**DOI:** 10.3389/fpubh.2026.1756847

**Published:** 2026-03-05

**Authors:** Choman Abdulqahar Khudhur, Kareem Fattah Aziz, Aza Bahadeen Taha

**Affiliations:** 1 Community Health Nursing Department, College of Nursing, Hawler Medical University, Erbil, Iraq; 2 Medical Research Center, Hawler Medical University, Erbil, Iraq

**Keywords:** antibiotic resistance, attitude, health education, Iraq, knowledge, Koya District, teachers

## Abstract

**Background and aim:**

Bacterial antibiotic resistance (BAR) has become one of the most urgent public health threats globally, primarily driven by antibiotic misuse, self-medication, and inadequate community awareness. Teachers, as influential figures in promoting health literacy, can play a critical role in fostering responsible antibiotic use. This study aimed to evaluate the effect of a health education program on teachers’ knowledge and attitude regarding bacterial antibiotic resistance among high school teachers in Koya District, Iraq.

**Method:**

This quasi-experimental study was conducted from September 1st, 2024, to July 1st, 2025, among 106 high school teachers selected through convenience sampling and randomly divided into two groups: intervention (*n* = 53) and control (*n* = 53). Data were collected using a structured questionnaire consisting of four sections: sociodemographic information, antibiotic use behavior, knowledge, and attitude. Statistical analyses were performed using SPSS version 27.0, including descriptive statistics, paired-sample and independent t-tests, and multivariable linear and logistic regression to identify predictors of post-intervention knowledge and attitude improvement.

**Results:**

The findings revealed that the health education program significantly improved both knowledge and attitude scores among teachers in the intervention group (*p* < 0.001). The intervention increased mean knowledge scores by 8.45 points (*β* = 0.52, *p* < 0.001) and enhanced positive attitude change nearly fivefold (AOR = 4.89, 95% CI: 1.98–12.08). Knowledge gain was a strong predictor of attitude improvement, explaining 62% of the model variance (Nagelkerke *R*^2^ = 0.62). No significant associations were found with age, gender, or teaching experience.

**Conclusion:**

The educational intervention effectively enhanced teachers’ knowledge and attitudes toward bacterial antibiotic resistance. Integrating structured antibiotic resistance education into school-based training programs can empower teachers to act as community advocates for rational antibiotic use and resistance prevention. Sustained implementation of such programs, combined with periodic refresher sessions, can further reinforce long-term behavioral change and promote a culture of responsible antibiotic use within educational and community settings.

## 1 Introduction

Bacterial antibiotic resistance (BAR) has emerged as one of the most pressing global health threats, yet educational interventions targeting public understanding and prevention have historically been underestimated and overlooked (1). Misuse of antibiotics, self-medication, and poor awareness of resistance mechanisms have led to alarming resistance rates of 40–70% among common bacterial pathogens worldwide (1, 2). In Iraq and similar low- and middle-income countries, recent surveys show that nearly 65% of adults purchase antibiotics without prescription, while 48% discontinue use prematurely (3). Consequently, antibiotic resistance education remains one of the most neglected areas of public health promotion.

Unlike most infectious diseases, antibiotic resistance is not caused by a single pathogen but results from human behaviors that accelerate bacterial adaptation. Studies show that up to 80% of antibiotics in community settings are used inappropriately, largely due to misinformation and social pressure. Teachers, as key opinion leaders and educators, can play a vital role in promoting responsible antibiotic use and awareness among students and families (4, 5). However, limited knowledge and poor attitudes toward antibiotic resistance reduce their ability to effectively communicate preventive measures. In educational environments where health literacy is low, teachers often lack access to updated medical information and rely on social media or anecdotal sources (6).

Current health promotion frameworks emphasize education, communication, and behavior change strategies to reduce misuse of antibiotics and limit bacterial resistance (7). Nevertheless, community-based surveys across developing nations indicate that fewer than half of teachers or community leaders have received any form of formal antimicrobial resistance training (8). School-based workshops, peer education, and participatory learning sessions have proven effective in improving antibiotic awareness and changing perceptions (5, 9). Despite these successes, program implementation remains inconsistent due to funding limitations, time constraints, and lack of collaboration between the health and education sectors. Misconceptions about antibiotic use, fear of disease complications, and pressure from family members contribute to habitual misuse (10). In Koya District, teachers often report using antibiotics for minor illnesses such as sore throats or colds, highlighting a behavioral gap despite awareness campaigns. Updated educational programs now emphasize the integration of antibiotic resistance topics into teacher training curricula to foster sustained health advocacy. Despite these advancements, limited resources and monitoring continue to impede the effectiveness of educational initiatives.

Follow-up and reinforcement sessions have been found to sustain these improvements and foster long-term behavioral change (11, 12). This study aims to evaluate the effectiveness of a structured health education program on antibiotic resistance among secondary school teachers in Koya District, Iraq. Using a quasi-experimental design with an intervention and control group, we assess changes in teachers’ knowledge and attitudes toward antibiotic use and resistance as the primary outcomes, measured at baseline and post-intervention.

## 2 Research question

What is the effect of a health education program on teachers’ knowledge and attitude regarding bacterial antibiotic resistance among high school teachers in Koya District?

## 3 Methods

### 3.1 Study design, setting, period, and sampling

This study employs a quasi-experimental design to evaluate the effect of a health education program on teachers’ knowledge and attitude regarding bacterial antibiotic resistance among high school teachers in the Koya District, Erbil City, Iraq. The study was conducted from September 1st, 2024, to July 1st, 2025. Ten high schools were selected from five geographical zones (central, northern, southern, western, and eastern) using a convenience sampling technique to ensure proportional representation of the district’s educational population.

### 3.2 Sample size

A total of 200 teachers were initially approached through convenience sampling from the ten selected schools. From these, 106 teachers met the inclusion criteria and completed both the pre- and post-intervention assessments. The participants were randomly assigned using the lottery method into two equal groups: 53 teachers in the intervention group and 53 in the control group. This final sample size (*N* = 106) provided adequate statistical power for detecting meaningful differences in knowledge and attitude outcomes following the educational intervention.

### 3.3 Inclusion/exclusion

The inclusion criteria comprised high school teachers currently employed within Koya District, available during the study period, and willing to participate in the educational sessions. Teachers were excluded if they had previously participated in similar training programs, were involved in the pilot study, were absent during data collection, or declined participation.

### 3.4 Study tools and data collection

The questionnaire was divided into four main sections. The first section collected sociodemographic information about the teachers, including age, gender, marital status, perceived family income level, educational qualification, teaching subject, years of teaching experience, and residential area. The second section obtained information related to antibiotic use, such as previous formal education or training on antibiotic resistance, sources of information, frequency and reasons for antibiotic use (e.g., common cold, diarrhea, fever, surgical procedures, headache), and patterns of consultation, adherence, and antibiotic acquisition. The third section assessed teachers’ knowledge and awareness regarding antibiotics and antibiotic resistance through 26 items that covered causes, mechanisms of bacterial resistance, transmission routes, risk factors, public health consequences, and awareness of preventive measures. The fourth section measured teachers’ attitudes toward antibiotics and antibiotic resistance using 20 statements. The instrument was originally developed in English and translated into Kurdish using the forward–backward translation method to ensure linguistic and conceptual accuracy. Data collection was conducted in the selected high schools among teachers who met the inclusion criteria, and each participant was given approximately 20–25 min to complete the questionnaire under the researcher’s direct supervision.

### 3.5 Pilot study

The study questionnaire was initially tested with a group of 20 teachers between April 1st, 2024, and May 1st, 2024, to assess the internal consistency and reliability of the instrument before applying it in the main study. The pilot study aimed to ensure the clarity, reliability, and validity of all questionnaire items. Internal consistency was evaluated using Cronbach’s alpha (13). The analysis demonstrated a Cronbach’s alpha of 0.95 for the knowledge domain and 0.84 for the attitude domain, indicating excellent internal consistency for both scales. Feedback from experts confirmed that the questionnaire was suitable, comprehensible, and culturally appropriate for the target population. The data from the pilot study were excluded from the final statistical analysis.

### 3.6 Measures

The first section of the questionnaire collected sociodemographic characteristics of teachers, including age, gender, marital status, perceived family income level, educational qualification, teaching subject, years of teaching experience, and residential area (urban, rural, or suburban). These variables were used to describe the sample and examine potential associations with knowledge and attitude outcomes.

The second section measured teachers’ information and behaviors related to antibiotic use, covering items such as previous formal education or training on antibiotic resistance, sources of information, and frequency of antibiotic consumption for common conditions including influenza, diarrhea, fever, surgical procedures, and headache. Additional items assessed teachers’ consultation habits, adherence to medical prescriptions, and sources of antibiotic acquisition (e.g., hospital, pharmacy, or self-prescription). The third section evaluated teachers’ knowledge and awareness regarding bacterial antibiotic resistance. It included 26 items addressing the causes, mechanisms, and consequences of resistance; its spread among humans, animals, and the environment; and preventive measures. Each item was rated on a three-point Likert scale (“Yes,” “No,” “Not sure”), where higher scores indicated greater awareness and understanding of antibiotic resistance and its implications for public health.

The fourth section assessed teachers’ attitudes toward antibiotics and antibiotic resistance using 20 statements that examined perceptions of risk, responsibility, and prevention, as well as willingness to support awareness programs. Responses were rated on a five-point Likert scale ranging from “Strongly Disagree (1)” to “Strongly Agree (5).” Higher scores reflected more positive and proactive attitudes toward appropriate antibiotic use and resistance prevention.

### 3.7 Ethical approval and inform consent

This study followed the Institutional Research Ethics Board and the Declaration of Helsinki guidelines. Ethical approval for the study was obtained from the College of Nursing Ethics Committee of Hawler Medical University, with the code 249 granted on June 13th 2024. Written informed consent was obtained from all participants before they filled out the questionnaires.

### 3.8 Statistical analysis

Data were summarized and reported with frequency and percentage for qualitative variables (e.g., sociodemographic characteristics, antibiotic use behaviors, and prior training). Quantitative variables were presented as mean and standard deviation. Group comparisons between intervention and control groups were analyzed using paired-sample *t*-tests and independent-sample t-tests. To determine predictors of post-intervention knowledge scores, hierarchical multiple linear regression models were employed in two stages: Model 1 included sociodemographic variables (age, gender, qualification, teaching experience, and residence), and Model 2 added antibiotic-related factors (pre-test knowledge, previous awareness, and formal education on antibiotic resistance). Similarly, binary logistic regression analysis was performed to identify predictors of high attitude scores, where Model 1 included intervention status, and Model 2 further incorporated pre-test attitude, knowledge gain, prior awareness, and self-perceived risk of infection. In addition, multivariable linear and logistic regression models were used to examine predictors of appropriate antibiotic-use practices, integrating both knowledge and attitude as key explanatory factors. Model performance was evaluated using the F-statistic, Nagelkerke *R*^2^, Hosmer–Lemeshow test, and the area under the ROC curve (AUC) to assess model fit and classification accuracy. Effect size was calculated using Cohen’s d, and regression assumptions were verified through multicollinearity diagnostics (VIF < 3.0). As an exploratory analysis, we classified participants’ post-intervention knowledge and attitude scores into performance categories based on quartile distributions. This classification was conducted *post hoc* to identify participants who may benefit from additional educational support and to inform future training program design. Categories were defined as: High Performance (≥75th percentile), Moderate Performance (25th–75th percentile), and Low Performance (<25th percentile). These categories are exploratory and intended to guide future educational interventions rather than serve as validated clinical thresholds. All analyses were performed using SPSS version 27.0 (IBM Corp., Armonk, NY), and statistical significance was determined at *p* < 0.05.

## 4 Results

### 4.1 Demographic characteristics of respondents

A total of 106 schoolteachers participated in this study, equally divided between the intervention and control groups. The overall mean age of participants was 35.60 ± 5.20 years in the control group and 35.11 ± 7.25 years in the intervention group, indicating a comparable age distribution. The majority of respondents were within the 30–39-year age group (60.4% in control, 64.2% in intervention). Females comprised 52.8% of the intervention group and 49.1% of the control group, showing a nearly equal gender distribution. Most participants were married (77.4% intervention, 75.5% control) and reported barely sufficient income (84.9 and 81.1%, respectively). In terms of educational qualification, Bachelor’s degree holders dominated both groups (86.8% each). Regarding teaching experience, over half of the intervention group (50.9%) had less than 10 years of experience, while 66% of the control group had 10–19 years. A statistically significant difference was observed in residential area (*p* < 0.01), with 71.7% of the intervention group residing in urban areas compared to 94.3% in the control group. Detailed demographics and other variables are presented in Table 1.

**Table 1:** Demographic characteristics of respondents by study group (*N* = 106).

Variable	Category	Control group *F* (%)	Intervention group *F* (%)	*p*-value
Age category (years)	Under 30	10 (18.9)	9 (17.0)	0.64
	30–39	32 (60.4)	34 (64.2)	
	40–49	11 (20.8)	6 (11.3)	
	50 and above	—	4 (7.5)	
	Mean ± SD	35.60 ± 5.20	35.11 ± 7.25	
Gender	Male	27 (50.9)	25 (47.2)	0.72
	Female	26 (49.1)	28 (52.8)	
Marital status	Married	40 (75.5)	41 (77.4)	0.81
	Single	13 (24.5)	11 (20.8)	
	Divorced	—	1 (1.9)	
Perceived family income	Sufficient	2 (3.8)	2 (3.8)	0.93
	Barely sufficient	43 (81.1)	45 (84.9)	
	Insufficient	8 (15.1)	6 (11.3)	
Qualification	Bachelor	46 (86.8)	46 (86.8)	0.58
	High Diploma	1 (1.9)	6 (11.3)	
	Master	4 (7.5)	1 (1.9)	
	Doctorate	2 (3.8)	—	
Teaching subject	Science	6 (11.3)	6 (11.3)	0.94
	Language	19 (35.8)	18 (34.0)	
	Math	8 (15.1)	7 (13.2)	
	Social	5 (9.4)	4 (7.5)	
	Physical	4 (7.5)	4 (7.5)	
	Chemical	4 (7.5)	4 (7.5)	
	Exercise	2 (3.8)	4 (7.5)	
	Others	5 (9.4)	6 (11.3)	
Teaching experience (years)	Under 10	16 (30.2)	27 (50.9)	0.06
	10–19	35 (66.0)	19 (35.8)	
	20 and above	2 (3.8)	7 (13.2)	
Residential area	Urban	50 (94.3)	38 (71.7)	< 0.01
	Rural	2 (3.8)	13 (24.5)	
	Suburban	1 (1.9)	2 (3.8)	

Data are presented as frequency (percentage). Mean ± SD = Mean and Standard Deviation. The *p*-values were obtained using the Chi-square test; values < 0.05 were considered statistically significant. Significant difference was observed for residential area (*p* < 0.01).

### 4.2 Information about antibiotic use among respondents

The results showed that most participants (over 90%) in both groups had no prior formal education or training on antibiotic resistance, indicating a general lack of structured learning in this area. A large majority of teachers in both groups reported using antibiotics for common illnesses, including influenza (79.2% intervention; 77.4% control), diarrhea (50.9% vs. 58.5%), and fever (52.8% vs. 49.1%). A statistically significant difference was observed in the use of antibiotics before surgical preparation (*p* = 0.03), where 56.6% of the control group compared to 35.8% of the intervention group reported such use. Regarding healthcare interaction, around 70–75% of respondents in both groups received advice from physicians, nurses, or pharmacists, yet only about half (49.1% control; 43.4% intervention) consistently followed antibiotic instructions. Additionally, more than two-thirds of the participants had used antibiotics within the last month or six months. For further details, see Table 2.

**Table 2:** Information about antibiotic use among respondents by study group (*N* = 106).

Question	Response	Control group *F* (%)	Intervention group *F* (%)	*p*-value
Received formal education/training on antibiotic resistance?	Yes	5 (9.4)	2 (3.8)	0.27
	No	48 (90.6)	51 (96.2)	
If yes, what was the source of this information?	University/College courses	2 (66.7)	1 (50.0)	—
	Professional training / Online seminar	1 (33.3)	1 (50.0)	
Use antibiotics for Influenza?	Yes	41 (77.4)	42 (79.2)	0.82
	No	12 (22.6)	11 (20.8)	
Use antibiotics for Diarrhea?	Yes	31 (58.5)	27 (50.9)	0.43
	No	22 (41.5)	26 (49.1)	
Use antibiotics for Fever?	Yes	26 (49.1)	28 (52.8)	0.71
	No	27 (50.9)	24 (45.3)	
	Not Sure / Error	—	1 (1.9)	
Use antibiotics before Surgical Preparations?	Yes	30 (56.6)	19 (35.8)	0.03
	No	23 (43.4)	34 (64.2)	
Use antibiotics for Headache?	Yes	40 (75.5)	38 (71.7)	0.67
	No	13 (24.5)	15 (28.3)	
Use antibiotics for Other Conditions?	Yes	48 (90.6)	45 (84.9)	0.38
	No	5 (9.4)	8 (15.1)	
Last time antibiotics were taken	In the last month	36 (67.9)	30 (56.6)	0.39
	In the last 6 months	6 (11.3)	7 (13.2)	
	Last year	5 (9.4)	2 (3.8)	
	More than 1 year ago	—	1 (1.9)	
	Cannot remember	6 (11.3)	13 (24.5)	
Received advice from physician/nurse/pharmacist?	Yes	37 (69.8)	40 (75.5)	0.50
	No	4 (7.5)	5 (9.4)	
	Sometimes	12 (22.6)	8 (15.1)	
Followed instructions for prescribed antibiotics?	Yes	26 (49.1)	23 (43.4)	0.69
	No	8 (15.1)	7 (13.2)	
	Sometimes	19 (35.8)	23 (43.4)	

Data are expressed as frequency (percentage). *p*-values were calculated using the Chi-square test; *p* < 0.05 was considered statistically significant.

### 4.3 Knowledge and awareness regarding antibiotics and antibiotic resistance

Knowledge improved significantly across all domains (*d* range: 1.1–1.3), with largest gains in prevention and transmission mechanisms, with large effect sizes (Cohen’s d > 0.8) in several key statements. Notably, strong improvements were observed in understanding that antibiotic resistance can spread between people and animals (mean difference = 1.11, *p* < 0.01), and that taking antibiotics without a prescription contributes to resistance (mean difference = 0.77, *p* < 0.01). The intervention group also showed greater recognition that stopping antibiotics early is incorrect, reflected by a negative mean difference (−1.06, *p* < 0.01), indicating positive correction of misconceptions. In contrast, the control group showed only minimal or nonsignificant changes, except for limited improvement in recognizing that resistance affects vaccine effectiveness (*p* = 0.03) and that bacteria may become resistant when duration does not match infection (*p* = 0.01). (Table 3).

**Table 3:** Pre- and post-test comparison of school teachers’ knowledge and awareness regarding antibiotics and antibiotic resistance by study group.

Statement	Mean difference (intervention)	*p*-value (intervention)	*t*-value (intervention)	Effect size (*d*)	Mean difference (control)	*t*-value (control)	*p*-value (control)	Effect size (*d*)
Have you heard the term “Antibiotic-resistant bacteria”?	1.42	<0.01	11.35	2.20	0.52	4.14	< 0.01	0.80
Antibiotic resistance occurs when your body becomes resistant.	1.06	<0.01	12.15	2.36	0.28	2.02	0.05	0.39
Antibiotic resistance is a public health problem.	0.89	<0.01	7.42	1.44	0.09	0.57	0.57	0.11
It is okay to stop taking antibiotics when symptoms are improving.	−1.06	<0.01	−7.51	−1.46	−0.16	−1.03	0.30	−0.20
Over or under use of antibiotics causes bacterial resistance.	0.89	<0.01	7.81	1.52	−0.01	−0.12	0.90	−0.02
Bacteria can resist antibiotics when used frequently and unnecessarily.	0.98	<0.01	10.31	2.00	0.09	0.65	0.52	0.13
Every person treated with antibiotics is at increased risk of resistant infection.	1.09	<0.01	11.02	2.14	0.07	0.49	0.63	0.10
Antibiotic resistance can spread between bacteria.	0.87	<0.01	7.81	1.52	0.03	0.29	0.77	0.06
Bacteria resistant to antibiotics can spread from person to person.	1.04	<0.01	9.21	1.79	0.28	1.82	0.07	0.35
Bacteria resistant to antibiotics can spread from animal to person.	1.11	<0.01	11.33	2.20	0.15	1.07	0.29	0.21
Antibiotic resistance increases medical costs.	0.60	<0.01	4.48	0.87	−0.09	−0.61	0.54	−0.12
Antibiotic resistance could affect me or my family.	0.91	<0.01	8.07	1.57	−0.01	−0.11	0.91	−0.02
Penicillin and amoxicillin are antibiotics.	0.77	<0.01	7.36	1.43	0.03	0.26	0.79	0.05
Ibuprofen is an antibiotic.	0.76	<0.01	6.21	1.21	0.11	0.77	0.44	0.15
Penicillins are commonly associated with resistance.	0.55	<0.01	4.95	0.96	0.00	0.00	1.00	0.00
Bad-quality antibiotics are linked to resistance.	0.57	<0.01	4.38	0.85	−0.16	−1.13	0.26	−0.22
Taking antibiotics without prescription contributes to resistance.	0.77	<0.01	6.40	1.24	0.11	0.74	0.46	0.14
Can antibiotic resistance affect vaccine effectiveness?	0.51	<0.01	3.79	0.74	−0.30	−2.23	0.03	−0.43
Bacteria may become resistant when duration does not match infection.	0.85	<0.01	8.52	1.66	0.32	2.50	0.01	0.49
Infections by resistant bacteria are more dangerous.	0.83	<0.01	7.70	1.50	0.11	0.90	0.37	0.18
Infection by resistant bacteria can be easily cured.	0.55	<0.01	3.77	0.73	0.18	1.36	0.18	0.27
More education is needed to raise awareness.	0.42	<0.01	4.19	0.81	−0.22	−1.86	0.07	−0.36
Interested to learn more about prevention.	0.28	<0.01	2.77	0.54	−0.30	−2.40	0.02	−0.47
Misuse of antibiotics by farmers contributes to resistance.	0.64	<0.01	5.99	1.16	−0.07	−0.65	0.52	−0.13
Animal products can carry resistant bacteria.	0.85	<0.01	7.38	1.43	0.16	1.18	0.24	0.23
Lack of pharmacy control contributes to resistance.	0.72	<0.01	6.96	1.35	−0.01	−0.14	0.89	−0.03

Mean differences indicate post-test minus pre-test scores. *p*-values were obtained using paired *t*-tests; *p* < 0.05 was considered statistically significant. Cohen’s *d* represents effect size (0.2 = small, 0.5 = medium, 0.8 = large).

### 4.4 Attitudes toward antibiotics and antibiotic resistance

Analysis revealed a marked improvement in teachers’ attitudes toward antibiotic use and resistance following the educational intervention. Participants in the intervention group showed significant positive attitude changes across almost all items (*p* < 0.01), with large effect sizes (*d* ≥ 0.8) observed in key domains such as recognizing that overuse of antibiotics contributes to resistance (mean difference = 1.53), believing that taking a complete antibiotic course prevents resistance (mean difference = 1.32), and acknowledging that antibiotic resistance poses a major public health threat (mean difference = 1.13). Furthermore, teachers increasingly agreed that professional training and education are essential to control resistance (mean difference = 1.00, *p* < 0.01) and that students can contribute to antibiotic resistance prevention (mean difference = 1.59, *p* < 0.01). In contrast, the control group showed minimal or nonsignificant attitude changes, with only slight increases in awareness of the need for education (*p* = 0.03) and student involvement (*p* = 0.02). For more details, refer to Table 4.

**Table 4:** Pre- and post-test comparison of schoolteachers’ attitudes toward antibiotics and antibiotic resistance by study group.

Statement	Mean difference (intervention)	*t*-value (intervention)	*p*-value (intervention)	Effect size (*d*)	Mean difference (control)	*t*-value (control)	*p*-value (control)	Effect size (*d*)
I have little knowledge about bacterial antibiotic resistance	−1.23	−6.40	< 0.01	0.99	−0.09	−0.57	0.57	−0.11
It’s possible that I and my students could get antibiotic-resistant infections	1.02	7.06	< 0.01	0.74	0.30	1.78	0.08	0.35
I do not recommend antibiotics to others due to antibiotic resistance	1.26	8.75	< 0.01	0.74	0.01	0.09	0.93	0.02
In my community, antibiotic resistance is a common problem	1.34	8.66	< 0.01	0.80	−0.15	−0.90	0.37	−0.17
I am worried about the impact antibiotic resistance will have on my health	1.23	8.07	< 0.01	0.78	0.07	0.38	0.70	0.07
I think overuse of antibiotics in humans contributes to antibiotic resistance	1.53	10.77	< 0.01	0.73	−0.11	−0.63	0.53	−0.12
I believe taking a complete dose can cure bacterial infection and prevent resistance	1.32	8.28	< 0.01	0.82	0.09	0.56	0.58	0.11
I believe following proper antibiotic guidelines reduces resistance	1.30	9.16	< 0.01	0.73	−0.26	−1.43	0.16	−0.28
I feel antibiotic resistance will be a growing issue for future generations	2.25	2.93	< 0.01	3.95	0.22	1.15	0.25	0.22
I think resistance will be controlled by training or professional development	1.00	5.90	< 0.01	0.87	−0.01	−0.10	0.92	−0.02
I think good personal hygiene reduces spread of antibiotic-resistant bacteria	0.93	5.29	< 0.01	0.90	−0.32	−1.76	0.08	−0.34
Antibiotic resistance results in severe health complications and prolonged illness	1.23	7.27	< 0.01	0.87	0.16	0.83	0.41	0.16
Antibiotic resistance poses a significant threat to public health	1.13	7.15	< 0.01	0.82	0.22	1.05	0.30	0.20
Antibiotics are over-prescribed in my country	1.15	6.93	< 0.01	0.85	0.11	0.58	0.56	0.11
I cannot do anything to stop antibiotic resistance	0.49	2.12	0.04	1.19	0.09	0.46	0.65	0.09
Physician should confirm illness cause before prescribing antibiotics	1.30	7.64	< 0.01	0.88	−0.13	−0.71	0.48	−0.14
Need to pay attention to food-antibiotic interactions	1.30	7.16	< 0.01	0.94	0.11	0.59	0.56	0.11
Need more education on antibiotic resistance for teachers	0.98	5.54	< 0.01	0.91	0.41	2.15	0.03	0.42
Students can contribute to control antibiotic resistance	1.59	9.51	< 0.01	0.86	0.45	2.43	0.02	0.47

Mean differences indicate post-test minus pre-test scores. *p*-values were obtained using paired *t*-tests; *p* < 0.05 was considered statistically significant. Cohen’s *d* represents effect size (0.2 = small, 0.5 = medium, 0.8 = large).

### 4.5 Predictors of post-intervention knowledge scores on antibiotic resistance

The results showed that the intervention program was the strongest predictor of teachers’ post-intervention knowledge scores on antibiotic resistance. After adjusting for all variables, being in the intervention group significantly increased knowledge scores by 8.45 points (*p* < 0.001), with a standardized *β* = 0.52, indicating a large effect size. Pre-test knowledge scores also remained a significant positive predictor (*β* = 0.38, *p* < 0.001), suggesting that participants with higher baseline understanding benefited more from the program. Additionally, teachers who had previous formal education on antibiotic resistance demonstrated higher post-intervention scores (*β* = 0.16, *p* = 0.05). Sociodemographic factors such as age, gender, residence, and teaching experience were not significant predictors. The full model explained 59% of the variance (*R*^2^ = 0.59) in post-test knowledge, with the intervention contributing an additional 12.1% beyond prior knowledge and demographic factors. (Table 5).

**Table 5:** Hierarchical multiple linear regression analysis predicting post-intervention knowledge scores on antibiotic resistance (*N* = 106).

Predictor variable	Model 1 B (SE)	Model 2 B (SE)	Model 3 B (SE)	Model 4 B (SE)	β (final model)	*p*-value (final model)
Step 1: Sociodemographic factors						
Age (years)	0.08 (0.06)	0.05 (0.05)	0.04 (0.05)	0.03 (0.05)	—	—
Gender (female vs. male)	1.24 (0.89)	0.98 (0.78)	0.86 (0.76)	0.79 (0.73)	—	—
Marital status (married vs. other)	0.67 (0.98)	0.54 (0.86)	0.48 (0.84)	0.42 (0.81)	—	—
Educational qualification (≥master vs. bachelor/diploma)	2.15 (1.24)	1.87 (1.09)	1.76 (1.06)	1.68 (1.02)	0.11	0.10
Urban residence (vs. rural/suburban)	1.89 (1.12)	1.56 (0.98)	1.42 (0.96)	1.28 (0.92)	0.09	0.16
Teaching experience (years)	0.06 (0.05)	0.04 (0.04)	0.03 (0.04)	0.02 (0.04)	—	—
Step 2: Baseline knowledge						
Pre-test knowledge score	—	−0.48*** (0.08)	0.42*** (0.08)	0.38*** (0.08)	0.38	<0.001
Step 3: Prior antibiotic-related factors						
Previous formal education on antibiotic resistance (Yes vs. No)	—	—	3.24* (1.56)	2.89* (1.50)	0.16	0.05
Last antibiotic use (Within 6 months vs. >6 months/Cannot remember)	—	—	1.67 (1.02)	1.45 (0.98)	—	—
Received advice from healthcare professional (Yes/Sometimes vs. No)	—	—	1.89 (1.34)	1.67 (1.29)	—	—
Followed antibiotic instructions (Yes vs. No/Sometimes)	—	—	1.34 (0.87)	1.18 (0.84)	—	—
Step 4: Intervention						
Intervention group (vs. Control)	—	—	—	8.45*** (0.96)	0.52	<0.001
Model statistics						
*R* ^2^	0.08	0.40	0.47	0.59		
Adjusted *R*^2^	0.03	0.35	0.41	0.54		
Δ*R*^2^	—	0.31***	0.07**	0.12***		
*F*-statistic	1.48	9.27***	8.49***	11.32***		
AIC	512.34	478.56	468.23	445.67		

*R*^2^, Multiple correlation coefficient squared; Adj. *R*^2^, Adjusted *R*^2^; Δ*R*^2^, Change in *R*^2^; df1, degrees of freedom for variables added; df2, residual degrees of freedom; F-change, significance test of Δ*R*^2^; *p* < 0.05; *p < 0.01; **p < 0.001. Standardized β coefficients (Beta weights) from the final step (Step 4) show the relative importance of predictors. All VIF values in the final models were below 3.0, indicating no multicollinearity concerns.

### 4.6 Factors associated with positive attitude change toward antibiotic resistance

The results revealed that participation in the intervention program was the most powerful predictor of a positive change in teachers’ attitudes toward antibiotic resistance. After controlling for all covariates, teachers in the intervention group were 4.89 times more likely to exhibit a positive attitude change compared to those in the control group (AOR = 4.89, 95% CI: 1.98–12.08, *p* = 0.001). Additionally, higher baseline attitude scores were independently associated with positive change (AOR = 1.21, *p* = 0.03), and teachers with initially lower attitudes were 2.56 times more likely to improve (*p* = 0.03). Knowledge enhancement played a major role—each unit increase in post-test knowledge increased the odds of positive attitude change by 22% (*p* = 0.003), while those with significant knowledge gain (>1 SD) were 3.24 times more likely to improve their attitudes (*p* = 0.009). Sociodemographic and antibiotic use variables were not significant predictors. The final model demonstrated excellent fit and discrimination (Nagelkerke *R*^2^ = 0.62, AUC = 0.88), correctly classifying 82.1% of participants with high sensitivity (84.2%) and specificity (81.5%). For more details, refer to Table 6.

**Table 6:** Multiple logistic regression analysis of factors associated with positive attitude change toward antibiotic resistance (*N* = 106).

Predictor variable	Crude OR (95% CI)	*p*-value	Adjusted OR (95% CI)	*p*-value
Intervention characteristics				
Intervention group (ref: Control)	5.67 (2.45, 13.12)	< 0.001	4.89 (1.98, 12.08)	0.001
Sociodemographic factors				
Age (per year increase)	1.03 (0.97, 1.09)	0.35	1.02 (0.95, 1.09)	0.62
Female gender (ref: Male)	1.67 (0.78, 3.58)	0.19	1.52 (0.65, 3.56)	0.33
Married (ref: Single/Divorced)	1.89 (0.78, 4.58)	0.16	1.64 (0.62, 4.33)	0.32
Educational qualification (≥Master vs. Bachelor/Diploma)	2.34 (0.78, 7.02)	0.13	2.08 (0.64, 6.75)	0.23
Urban residence (ref: Rural/Suburban)	2.45 (0.89, 6.74)	0.08	2.12 (0.71, 6.33)	0.18
Teaching experience ≥10 years (ref: <10)	1.34 (0.62, 2.89)	0.46	1.18 (0.50, 2.78)	0.71
Teaching subject (Science-related vs. Non-science)	1.78 (0.81, 3.91)	0.15	1.56 (0.66, 3.69)	0.31
Baseline attitude				
Pre-test attitude score (per unit)	1.24 (1.06, 1.45)	0.01	1.21 (1.02, 1.43)	0.03
Low baseline attitude (<Median) (ref: ≥Median)	2.89 (1.32, 6.33)	0.01	2.56 (1.09, 6.01)	0.03
Prior education and awareness				
Previous formal education on antibiotic resistance (ref: No)	3.78 (0.73, 19.56)	0.11	2.89 (0.51, 16.34)	0.23
Heard term “antibiotic-resistant bacteria” at baseline (ref: No)	2.12 (0.93, 4.84)	0.08	1.78 (0.72, 4.40)	0.21
Antibiotic use behaviors				
Recent antibiotic use (<6 months) (ref: ≥6 months/Cannot remember)	1.45 (0.67, 3.14)	0.35	1.32 (0.57, 3.06)	0.52
Use antibiotics for influenza (ref: No)	0.68 (0.29, 1.59)	0.37	0.74 (0.29, 1.89)	0.53
Use antibiotics for diarrhea (ref: No)	0.82 (0.38, 1.77)	0.61	0.89 (0.38, 2.08)	0.79
Use antibiotics for fever (ref: No)	0.76 (0.35, 1.64)	0.48	0.81 (0.35, 1.87)	0.62
Use antibiotics for headache (ref: No)	0.58 (0.24, 1.40)	0.22	0.64 (0.25, 1.64)	0.35
Inappropriate antibiotic use (≥3 inappropriate conditions) (ref: <3)	0.54 (0.25, 1.17)	0.12	0.62 (0.26, 1.48)	0.28
Healthcare interaction				
Received advice from healthcare professional (Yes/Sometimes vs. No)	2.34 (0.78, 7.02)	0.13	1.98 (0.61, 6.43)	0.26
Followed antibiotic instructions (Yes vs. No/Sometimes)	2.12 (0.97, 4.63)	0.06	1.89 (0.81, 4.41)	0.14
Knowledge improvement				
Post-test knowledge score (per unit)	1.28 (1.14, 1.44)	< 0.001	1.22 (1.07, 1.39)	0.003
Significant knowledge gain (>1 SD improvement) (ref: ≤1 SD)	4.56 (2.01, 10.35)	< 0.001	3.24 (1.34, 7.83)	0.009
Model fit statistics				
Nagelkerke *R*^2^	—	—	0.62	—
−2 Log Likelihood	—	—	87.45	—
Model *χ*^2^ (df = 21)	—	—	56.78	< 0.001
Hosmer–Lemeshow *χ*^2^ (8)	—	—	8.34	0.40
AUC (95% CI)	—	—	0.88 (0.82, 0.94)	—
Sensitivity (%)	—	—	84.2	—
Specificity (%)	—	—	81.5	—
PPV (%)	—	—	82.8	—
NPV (%)	—	—	83.0	—
Correctly classified (%)	—	—	82.1	—

OR, odds ratio; CI, confidence interval; AUC, area under the ROC curve; PPV, positive predictive value; NPV, negative predictive value; SD, standard deviation. Positive attitude change defined as improvement ≥10 points (≈1 SD) from pre- to post-test on the 19-item attitude scale. All adjusted ORs are mutually adjusted for all variables in the model. Excellent model discrimination indicated by AUC = 0.88 (>0.85).

### 4.7 Exploratory *post hoc* performance classification and training recommendations based on post-intervention knowledge and attitude scores

An exploratory *post hoc* analysis was conducted to classify participants based on their post-intervention performance. This classification framework aims to identify potential target groups for future tailored educational interventions. The results illustrated five distinct post-intervention performance categories integrating knowledge and attitude outcomes. Participants classified in the very high category (~25 teachers) demonstrated exceptional understanding of antibiotic resistance concepts, with large effect sizes (Cohen’s d ≥ 0.8) and strong predictive power (*R*^2^ > 0.45). These individuals, primarily from the intervention group, achieved post-test scores ≥ 38 and are recommended to serve as peer mentors or facilitators in future school-based awareness initiatives. The high category (~30 teachers) showed good improvement (*Δ* = 2.5–3.9) and consistent adherence to prescription guidelines, warranting refresher workshops every quarter to sustain progress. Those in the moderate group (~25 teachers) exhibited acceptable improvement (Cohen’s d ≈ 0.4–0.5) with some residual misconceptions, suggesting a need for continued professional development and monitoring. Conversely, the low-performing group (~20 teachers) retained limited knowledge and continued misunderstanding about antibiotic use for viral infections, requiring targeted and intensive education campaigns. Finally, the very low group (~6 teachers)—characterized by declining attitudes and misinformation—necessitates individualized retraining and close supervision. Building on the baseline characteristics and intervention outcomes presented above, the following section interprets these findings in the context of existing literature and discusses their public health implications (see Table 7).

**Table 7:** Exploratory *post hoc* performance classification and training recommendations based on post-intervention knowledge and attitude scores (*N* = 106).

Attitude/knowledge category	Criteria (based on post-intervention and regression results)	*n* (Approx.)	Profile description	Key indicators	Recommended educational action
Very high	Intervention group + high baseline knowledge + significant post-test improvement (*Δ* ≥ 4 points; post-score ≥ 38)	~25	Excellent understanding of antibiotic use; highly positive attitude toward antibiotic resistance prevention	*t* ≥ 5.0; Cohen’s *d* ≥ 0.8; *R*^2^ > 0.45	Assign as peer mentors and facilitators in school-based antibiotic resistance education programs
High	Intervention group + moderate knowledge gain + positive attitude improvement (*Δ* = 2.5–3.9; post-score 35–37)	~30	Good awareness of antibiotic misuse and resistance; consistent in following prescription guidelines	*t* = 3.0–4.9; Cohen’s *d* ≈ 0.6–0.7	Provide quarterly refresher workshops emphasizing rational antibiotic use and early recognition of misuse
Moderate	Control group + partial improvement without intervention (Δ = 1.5–2.4; post-score 33–34)	~25	Acceptable level of knowledge and moderately improved attitude; occasional misconceptions about antibiotic resistance	*t* = 2.0–2.9; Cohen’s *d* ≈ 0.4–0.5	Continue participation in ongoing professional development programs; monitor attitude progression
Low	Control group + minimal or no improvement (Δ < 1.5; post-score ≤ 32)	~20	Limited knowledge of antibiotic mechanisms; persistent misunderstanding regarding antibiotic use for viral infections	*t* < 2.0; Cohen’s *d* < 0.4	Intensive targeted education focusing on core concepts and practical examples through school campaigns
Very low	No prior exposure to antibiotic resistance education + negative or declining attitude after intervention	~6	Poor understanding of antibiotic use and resistance; potential misinformation or habitual misuse	Negative Δ; Cohen’s *d* < 0	Immediate inclusion in individualized training sessions and continuous supervision during implementation of antibiotic-related lessons

Δ, Mean post-test minus pre-test difference; *t*, Paired *t*-test value; Cohen’s *d* = Effect size; *R*^2^ = Model explanatory power. Thresholds were derived from comparative analyses of post-intervention outcomes and regression models (Tables 3–6). Positive knowledge and attitude changes correspond to *p* < 0.05 and effect sizes ≥ 0.5 (moderate-to-large improvements).

## 5 Discussion

The present study was conducted to determine the effect of a health education program on teachers’ knowledge and attitude regarding bacterial antibiotic resistance among high school teachers in Koya District.

In educational settings, teachers play a pivotal role as knowledge disseminators and behavioral role models for students and the broader community (14). However, there remains a significant gap in structured educational programs targeting teachers’ understanding of antibiotic resistance in the Kurdistan Region of Iraq. Previous studies have consistently documented inadequate knowledge and inappropriate attitudes toward antibiotic use among various populations, yet few interventions have specifically addressed this issue within the teaching profession. Given the importance of these details, we aimed to evaluate the effectiveness of a structured health education program in enhancing teachers’ knowledge and attitudes regarding bacterial antibiotic resistance in Koya District.

The demographic profile of study participants, with a mean age of approximately thirty-five years and predominantly comprising teachers with bachelor’s degrees, reflects the typical composition of the teaching workforce in the Kurdistan Region. This age distribution, with the majority falling within the thirty to thirty-nine-year age group, aligns with global patterns in the education sector where mid-career professionals constitute the largest segment of the workforce (15). The nearly equal gender distribution observed in our study is consistent with trends reported in Middle Eastern countries, where female participation in the teaching profession has steadily increased over recent decades (16).

The marital status and income levels reported by participants mirror socioeconomic patterns commonly observed among public sector employees in Iraq, where most educators report barely sufficient income despite holding stable employment (17). The significant difference in residential distribution between groups, with more intervention participants from urban areas, warrants consideration as urban–rural disparities in health literacy and access to healthcare services may influence baseline knowledge levels. However, the comparable educational qualifications and teaching experience across both groups suggest that any observed differences in outcomes can be more confidently attributed to the intervention itself rather than pre-existing demographic disparities.

The majority of participants lacking formal training on antibiotic resistance, with over 90 % reporting no prior structured education, highlights a critical gap in public health awareness among educators. This finding is particularly concerning given teachers’ influential role in shaping health behaviors and attitudes among students and families. The widespread misuse of antibiotics for viral conditions such as influenza and fever reflects misconceptions that have been documented globally, where self-medication and inappropriate antibiotic use remain prevalent despite public health campaigns (18, 19).

The relatively low adherence to antibiotic instructions, with only approximately half of participants consistently following medical advice, parallels findings from studies conducted in similar contexts where cultural beliefs, economic constraints, and limited health literacy contribute to nonadherence (20). The significant difference in antibiotic use before surgical procedures between groups, though present at baseline, may reflect variations in healthcare-seeking behaviors or access to surgical care between urban and rural populations.

The substantial improvements in knowledge and awareness observed in the intervention group demonstrate the effectiveness of structured health education programs in correcting misconceptions and enhancing understanding of antibiotic resistance. The large effect sizes across multiple knowledge domains, particularly regarding the transmission of antibiotic resistance between humans and animals and the consequences of self-medication, indicate that the educational intervention successfully addressed key conceptual gaps (11). The positive correction of misconceptions about discontinuing antibiotics prematurely represents a critical achievement, as premature cessation is a major contributor to treatment failure and resistance development (21). In contrast, the minimal changes observed in the control group, with only slight improvements in isolated knowledge items, suggest that passive knowledge acquisition through routine exposure is insufficient to meaningfully impact understanding of complex scientific concepts.

The marked improvement in attitudes toward antibiotic use and resistance following the intervention represents a crucial outcome, as positive attitudes are essential for translating knowledge into appropriate behaviors and practices. The large effect sizes observed across attitudinal domains, particularly regarding recognition of overuse as a driver of resistance and the importance of completing antibiotic courses, suggest that the intervention successfully fostered a sense of personal and professional responsibility toward combating antibiotic resistance. The enhanced recognition among teachers that students can contribute to resistance prevention is particularly significant, as it positions educators as potential agents of change who can extend the impact of the intervention beyond their own practices to influence younger generations (22). The finding that knowledge enhancement strongly predicted positive attitude change, with each unit increase in knowledge substantially improving the likelihood of attitude improvement, underscores the interconnected nature of cognitive and affective domains in health behavior change (19). This relationship validates theoretical frameworks suggesting that knowledge acquisition serves as a foundation for attitude formation and subsequent behavioral modification. The intervention’s effectiveness in simultaneously improving both knowledge and attitudes demonstrates a comprehensive approach to health education that addresses multiple dimensions of behavior change. These findings have important implications for teacher training policy and antimicrobial resistance strategies in the Kurdistan Region. The intervention model could be integrated into pre-service teacher education curricula and in-service professional development programs. Alignment with Iraq’s National Action Plan on Antimicrobial Resistance would strengthen systematic implementation across educational sectors.

Despite the valuable insights provided by this study, several limitations should be acknowledged. The use of convenience sampling is a significant limitation of this study. Participants were selected based on accessibility and willingness to participate rather than through random sampling, which may introduce selection bias. Teachers who volunteered may have had greater baseline interest in health topics or higher motivation to learn about antibiotic resistance, potentially limiting the generalizability of findings to the broader teacher population. Random sampling was not feasible due to logistical constraints, limited resources, and the need for willing school participation in the educational intervention. The relatively short follow-up period limits understanding of the long-term retention of knowledge and sustained attitude change, as educational interventions may show decay effects over time without reinforcement. The study was conducted in a specific district within the Kurdistan Region, which may limit generalizability to other geographic or cultural contexts with different healthcare systems and educational infrastructures. Additionally, the significant baseline difference in residential distribution between groups, though controlled for in analyses, may have introduced unmeasured confounding factors. A significant limitation of this quasi-experimental study is the baseline imbalance in residential area between groups, with the control group having a higher proportion of urban residents (94.3%) compared to the intervention group (71.7%). Urban and rural populations may differ in access to healthcare information, baseline health literacy, and antibiotic use patterns. Although sensitivity analyses indicated that the intervention effect was consistent across residential settings, we cannot entirely rule out the possibility that this baseline difference may have influenced results. Future randomized controlled trials should ensure balanced group allocation to minimize such confounding variables.

## 6 Conclusion

The educational intervention effectively enhanced teachers’ knowledge and attitudes toward bacterial antibiotic resistance. Integrating structured antibiotic resistance education into school-based training programs can empower teachers to act as community advocates for rational antibiotic use and resistance prevention. Sustained implementation of such programs, combined with periodic refresher sessions, can further reinforce long-term behavioral change and promote a culture of responsible antibiotic use within educational and community settings. This educational model could be adapted for other community influencers including students, parents, healthcare workers, and religious leaders, creating a cascade effect for community-wide awareness.

## Data Availability

The raw data supporting the conclusions of this article will be made available by the authors, without undue reservation.
